# Micrografting Provides Evidence for Systemic Regulation of Sulfur Metabolism between Shoot and Root

**DOI:** 10.3390/plants10081729

**Published:** 2021-08-20

**Authors:** Ilaria Forieri, Rasha Aref, Markus Wirtz, Rüdiger Hell

**Affiliations:** 1 Centre for Organismal Studies, University of Heidelberg, 69120 Heidelberg, Germany; ilaria.forieri@gmail.com (I.F.); rasha_aref@agr.asu.edu.eg (R.A.); markus.wirtz@cos.uni-heidelberg.de (M.W.); 2 Department of Genetics, Faculty of Agriculture, Ain Shams University, Cairo 11241, Egypt

**Keywords:** organ communication, sulfur homeostasis, sulfate transporter, sulfite reductase, *O*-acetylserine, grafting

## Abstract

The uptake of sulfate by roots and its reductive assimilation mainly in the leaves are not only essential for plant growth and development but also for defense responses against biotic and abiotic stresses. The latter functions result in stimulus-induced fluctuations of sulfur demand at the cellular level. However, the maintenance and acclimation of sulfur homeostasis at local and systemic levels is not fully understood. Previous research mostly focused on signaling in response to external sulfate supply to roots. Here we apply micrografting of Arabidopsis wildtype knock-down *sir1-1* mutant plants that suffer from an internally lowered reductive sulfur assimilation and a concomitant slow growth phenotype. Homografts of wildtype and *sir1-1* confirm the hallmarks of non-grafted *sir1-1* mutants, displaying substantial induction of sulfate transporter genes in roots and sulfate accumulation in shoots. Heterografts of wildtype scions and *sir1-1* rootstocks and *vice versa*, respectively, demonstrate a dominant role of the shoot over the root with respect to sulfur-related gene expression, sulfate accumulation and organic sulfur metabolites, including the regulatory compound *O*-acetylserine. The results provide evidence for demand-driven control of the shoot over the sulfate uptake system of roots under sulfur-sufficient conditions, allowing sulfur uptake and transport to the shoot for dynamic responses.

## 1. Introduction

Sulfur metabolism from sulfate uptake to the formation of organic sulfur compounds is present in all plants as part of their autotrophic lifestyle. The model plant *Arabidopsis thaliana* is by far the best investigated species with respect to primary and secondary sulfur metabolism [1,2]. Uptake of inorganic sulfate (SO_4_^2−^) from the environment and its distribution throughout the plant is mediated by members of the sulfate transporter (SULTR) family [3]. The two genes encoding the plasmalemma localized high-affinity transporters SULTR1;1 and SULTR1;2 are almost exclusively expressed at the root surface. SULTR1 carries out the bulk of sulfate import, but the *SULTR1;1* gene is stronger induced during external sulfate limitation and, therefore, often used as a marker of sulfur nutritional status [4]. Other members of the SULTR family are functioning in the translocation of sulfate from root to shoot (group 2), the import into plastids (group 3) and export from vacuoles (group 4) [4,5]. Importantly, the assimilatory reduction of sulfate takes place in plastids of photoautotrophic and heterotrophic plant cells. It is initiated by ATP sulfurylase (ATPS, EC 2.7.7.4), forming adenosine 5´- phosphosulfate. APS reductase (APR, EC 1.8.4.9) then uses two electrons from glutathione (GSH) to catalyze the reduction of APS to sulfite (SO_3_^2−^). The latter is reduced to sulfide (S^2−^) by sulfite reductase (SiR, EC 1.8.7.1), which receives six electrons from either photosynthesis (green cells) or the oxidative pentose phosphate pathway (white cells). In the final reaction sequence, sulfide is inserted into cysteine, which acts as a hub for the distribution of reduced sulfur into the many different downstream products, such as methionine and GSH [6]. With respect to regulatory steps in the pathway, the uptake of sulfate into the cell is a prerequisite, but APR underlies complex transcriptional and post-translational control mechanisms in response to many different environmental stresses and dominates flux control in the pathway [1,7]. Interestingly, the step catalyzed by SiR is much less regulated but gains substantial control under sulfur starvation conditions [8]. Moreover, SiR is encoded by the only single-copy gene in sulfate assimilation of Arabidopsis [9], making this step a valuable tool for the investigation of primary sulfur metabolism [10,11,12]. 

Thus, nearly all cells of a plant possess the ability to assimilate sulfate, and accordingly, the respective organs show at least a partial autonomy with respect to assimilatory sulfate reduction. However, the bulk of sulfate is believed to be reduced by electrons from photosynthesis in leaves [1,2,13], implying the transport of sulfur is reduced by photosynthesis from shoot to the root. 

Here, the question of the systemic interaction of the shoots and roots with respect to the metabolic and transcriptomic remodeling under internal sulfur limitation was addressed. The typical response to sulfate deficiency in the soil in most plants consists in an early up-regulation of *Sultr1;1* and subsequently of many other genes [14,15]. Prolonged starvation results in chlorosis of older leaves and acclimatory differentiation of the root system [2]. How the shoots and roots communicate with respect to sulfate assimilation and growth control is largely unknown. In contrast to the well-investigated external depletion of sulfate, a grafting approach between Arabidopsis root and the shoot of wildtype and the *sir1-1* mutant was applied. The T-DNA insertion in the *sir1-1* mutant is located in the promoter region, causing lowered expression of the *SiR* gene and SiR protein. This causes a severe limitation of reduced sulfur for biosynthetic pathways and results in the constitutive up-regulation of root *Sultr1;1* expression and accumulation of sulfate in leaves [10,11,12]. This de-regulation of sulfur metabolism gives rise to a strong and easy-to-score growth retardation of the shoots and roots [12]. The mutant *sir1-2* is also a promoter insertion mutant, with 14% *SiR* transcript levels left compared to the wildtype and is seedling lethal in homozygote plants. In contrast, *sir1-1* has around 23% *SiR* transcript levels in mature leaves, concomitantly lowered SiR protein and enzyme activity levels and accordingly lowered levels in roots compared to the wildtype [11,12]. The somewhat weaker alleles *sir1-3* and *sir1-4* are also slightly growth retarded and have been shown to be sensitive to SO_2_ stress, pointing to further important functions of SiR in plants [16,17]. 

Thus, the *sir1-1* mutant has an intrinsic deficiency for reduced sulfur, making it the ideal complement to the external sulfate deficiency experiments for the investigation of systemic regulation of sulfur metabolism. The shoot or root was combined with the corresponding wildtype organ using micrografting to generate homo and heteromeric chimeric plants. The results demonstrate that the disruption of shoot sulfur assimilation is able to influence the regulation of sulfate uptake at the root level, the composition of primary sulfur compounds and root growth, but not vice versa. Thus, the grafting approach is a suitable tool to address systemic relationships and indicates that the shoot is in command of the autonomous sites of sulfur metabolism when external sulfur supply is not limiting.

## 2. Results

### 2.1. Phenotypical Analysis of Micrografted Wildtype and sir1-1 Arabidopsis Plants

**Figure 1:**
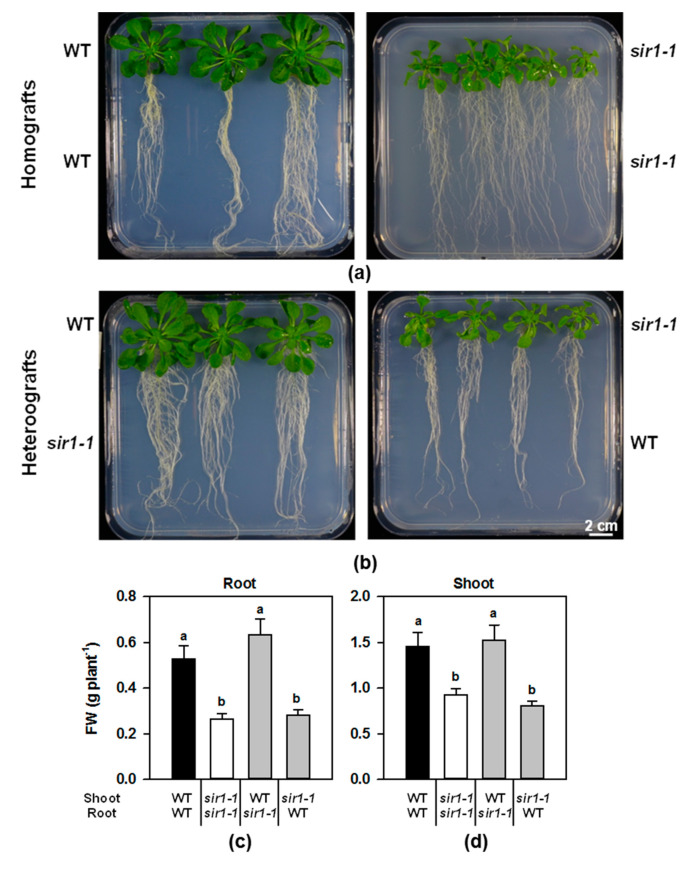
Phenotypic analysis of micrografted plants. (**a**,**b**) Phenotype of grafted plants five weeks after germination; selected representatives are shown for the four different grafting combinations. (**c**,**d**) Fresh weight (FW) of roots and shoots. Data represent mean and standard error and are modified according to [11] (*n* > 15). Different letters indicate statistically significant differences; *p* < 0.05, ANOVA followed by Dunn´s test; FW = fresh weight.

Micrografting was performed with 6–7-day-old wildtype (WT) and *sir1-1* seedlings to obtain chimeric plants (heterografts) that had the shoot of one genotype and the root of the other: WT shoot/*sir1-1* root (WT/*sir1-1*) and *sir1-1* shoot/WT root. As controls, homografts were included in each grafting experiment: WT shoot/WT root (WT/WT) and *sir1-1* shoot/*sir1-1* root (*sir1-1*/*sir1-1*). Grafted plants were harvested at the end of the fifth week after seed germination, and the fresh weight of rosette and roots were determined prior to gene expression and metabolite analyses. The results are modified from the data shown in [11]. The control homografts of WT/WT and *sir1-1*/*sir1-1* successfully reproduced the morphology and *SiR* depletion-induced slower growth that is observed for *sir1-1* lines grown in soil [12] (Figure 1a). The *sir1-1*/*sir1-1* plants were significantly smaller compared to WT/WT plants and accordingly had reduced fresh weights of roots (2-fold) and shoots (1.6-fold) (Figure 1c,d). The heterografts resulted in distinct phenotypes (Figure 1b): the WT/*sir1-1* combination showed a WT-like phenotype in the shoot and also in the root, whereas the *sir1-1*/WT combination resulted in plants that had *sir1-1*-like shoots and also roots. The phenotype of these heterografts was reflected by the measured fresh weights of both roots and shoot (Figure 1c,d). In fact, the WT/*sir1-1* combination showed no measurable difference in the fresh weight of both roots and shoots compared to the WT/WT homografts. The *sir1-1*/WT combination instead showed a significant decrease in the fresh weight of both roots and shoot (1.9-fold and 1.8-fold, respectively). 

From the phenotypic analysis of reciprocal *sir1-1* graftings, it is evident that the shoot imposes the growth phenotype to the root as shown previously [11]. However, it was unknown if this growth phenotype is exclusively a consequence of depleted SiR activity in shoots or also caused by misbalanced organ–organ communication via shoot-derived long distancing signaling. To address the potential impact of impaired shoot-sulfur reduction capacity on the S-uptake capacity/S-metabolism of the root, we determined steady-state transcript levels and key metabolites of the S-assimilation pathway in both organs of the homografts or heterografts of wildtype and *sir1-1*.

### 2.2. SiR, APR2 and SULTR1;1 Transcripts Are Differentially Expressed in Micrografted Plants

**Figure 2:**
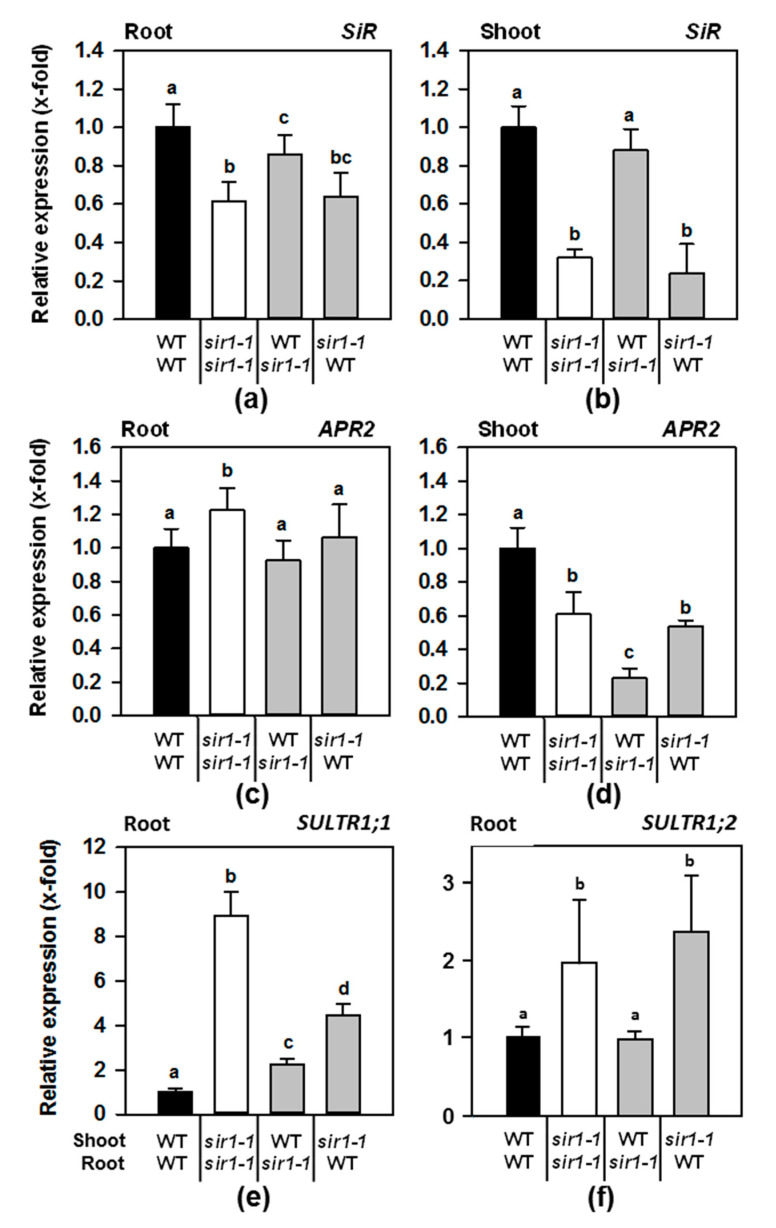
Steady-state transcript levels of key genes in the sulfur-assimilation pathway in micrografted plants. Transcript levels were determined by qRT-PCR using *TIP41*-like (At4g34270) as the reference gene in the roots (**a**,**c**,**e**,**f**) and shoots (**b**,**d**) of grafted plants. The level in control roots and shoots was set to 1, and the other values are expressed as x-fold of control. Data are shown as means ± SE (*n* = 4). Different letters indicate individual groups identified by multiple pairwise comparisons with a Holm–Sidak, one-way ANOVA (*p*, 0.05) followed by the Student–Newman–Keuls posthoc test.

The location of the T-DNA in the promotor region of the *sir1-1* allele causes different degrees of reduction of *SiR1* mRNA levels in different organs. Mature leaves and roots of the *sir1-1* mutant display an up to a 4-fold decrease in *SiR* transcript amount [11,12]. The micrografted plantlets very much reflected this differential expression pattern, confirming the suitability of the approach: in the roots of *sir1-1*/*sir1-1* homografts, *SiR* mRNA level was down to 60% and in the shoot, down to 30% compared to the WT/WT homografts (Figure 2a). Remarkably, in WT/*sir1-1* heterograft roots, the *SiR* expression was found to be decreased only by about 15% compared to the WT/WT combination, while in the roots of *sir1-1*/WT, the *SiR* mRNA level was decreased to the same extent as in *sir1-1*/*sir1-1* roots. This means that (1) the WT scion nearly reconstituted SiR expression in *sir1-1* root and (2) the *sir1-1* scion led to lowered SiR expression in WT root, indicating control from the shoot to the root. In contrast, the shoot SiR expression in the *sir1-1*/*sir1-1* autografts (Figure 2b) was downregulated 3.1-fold and unchanged in the WT/*sir1-1* graft combination while it was 4.2-fold down-regulated in the *sir1-1*/WT combination, excluding an effect on SiR expression from the root to the shoot.

The key regulated step in sulfate reduction during external sulfate deficiency is encoded by *APR2*, the major one of the three APR isoforms in Arabidopsis [18]. Depletion of SiR activity in the shoots of seven-week-old *sir1-1* plants compared to WT resulted in feedback inhibition of the *APR2* gene, while *APR1* and *APR3* transcription were unaffected [12]. However, in roots of hydroponically grown *sir1-1*, all *APR* isoforms were induced, suggesting that this *APR2* feedback inhibition might be controlled in an organ-specific manner [11]. Thus, the *APR2* transcript level was measured in both the roots and shoots of micrografted plants to assess whether the different tissues have an impact on the regulation of the response to constitutive internal sulfur deficiency (Figure 2b). 

*APR2* expression was upregulated 1.2-fold in the roots of *sir1-1*/*sir1-1* homografts compared to the WT/WT plants, whereas it was unchanged in the roots of the two hetero-graft combinations (Figure 2c). These findings demonstrate that *APR2* expression was not a subject of SiR depletion-induced feedback inhibition in roots, no matter if SiR was depleted in the shoot or the root itself. The *APR2* steady-state transcript levels in the shoot of *sir1-1*/WT and *sir1-1*/*sir1-1* were decreased to 60% of the wildtype level, which confirmed the previously shown *APR2* feedback inhibition in the shoots of soil-grown *sir1-1* [12]. These results suggest that the *APR2* feedback inhibition is a direct consequence of decreased SiR activity in the shoot and is restricted to the shoot (see above). Surprisingly, grafting of a *sir1-1* root to the WT shoot also caused substantial depletion of *APR2* transcript (Figure 2d, WT/*sir1-1*), albeit these plants grew similar to the wildtype (Figure 1).

One of the peculiarities of the T-DNA insertional mutant *sir1-1* is a constitutively enhanced expression of the high-affinity sulfate transporter gene *SULTR1;1* in roots of up to 10-fold [10], which results in increased sulfate accumulation in the shoot compared to the wildtype [10,12]. The homografts *sir1-1*/*sir1-1* indeed showed an 8.9-fold upregulation of *SULTR1;1* expression (Figure 2e). The WT/*sir1-1* grafting combination and the *sir1-1*/WT combination up-regulated *SULTR1;1* transcript level 2.2-fold and 4.4-fold, respectively. Analysis of the bulk uptake system encoded by *SULTR1;2* showed a similar pattern with enhanced expression in *sir1-1*/*sir1-1* and *sir1-1*/WT grafts, albeit at a lower level (Figure 2f). These results further support the hypothesis that deregulated sulfate assimilation in the shoot can influence the regulation of sulfate uptake and assimilation in the roots.

### 2.3. Shoot-Specific De-Regulation of Sulfate Assimilation Causes Specific Adaption of Major Nutrients in Root and Shoot of Micrografted Plants

**Figure 3:**
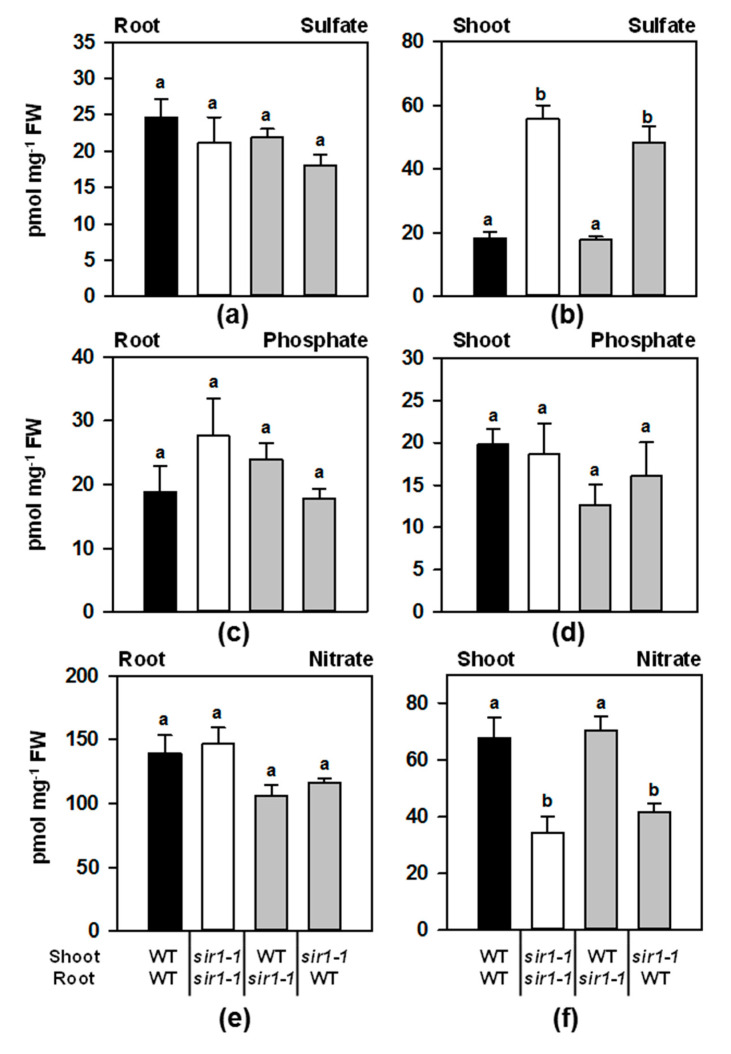
Steady-state levels of macronutrients in the roots and shoots of micrografted plants. Sulfate (**a**,**b**), phosphate (**c**,**d**) and nitrate (**e**,**f**) concentrations in roots (**a**,**c**,**e**) and shoots (**b**,**d**,**f**) of micrografted Arabidopsis plants. Data represent the means ± SE (*n* = 4). Different letters indicate individual groups identified by multiple pairwise comparisons with a Holm–Sidak, one-way ANOVA (*p*, 0.05) followed by the Student–Newman–Keuls posthoc test.

The *sir1-1* mutant plants show altered sulfate concentrations in the shoot compared to WT and simultaneously a disturbed nitrogen metabolism with a significant decrease of nitrate and of reduced nitrogen-containing compounds [12]. Sulfate and nitrate were quantified in micrografted plants together with phosphate to further dissect the differential contribution of root and shoot in the regulation of the responses to intrinsic sulfur starvation (Figure 3). 

Indeed, *sir1-1*/*sir1-1* plants confirmed the substantial accumulation of sulfate in the shoots of soil-grown *sir1-1* plants. This increase also occurred when SIR activity was depleted in shoots of *sir1-1*/WT grafts. However, the decrease of SiR in the roots of WT/*sir1-1* did not result in sulfate accumulation in the scion when compared to WT/WT (Figure 3b). The shoot-specific accumulation of sulfate correlated with the induction of the high-affinity sulfate uptake systems in roots if the scion had a *sir1-1*- background (Figure 2e,f). 

**Figure 4:**
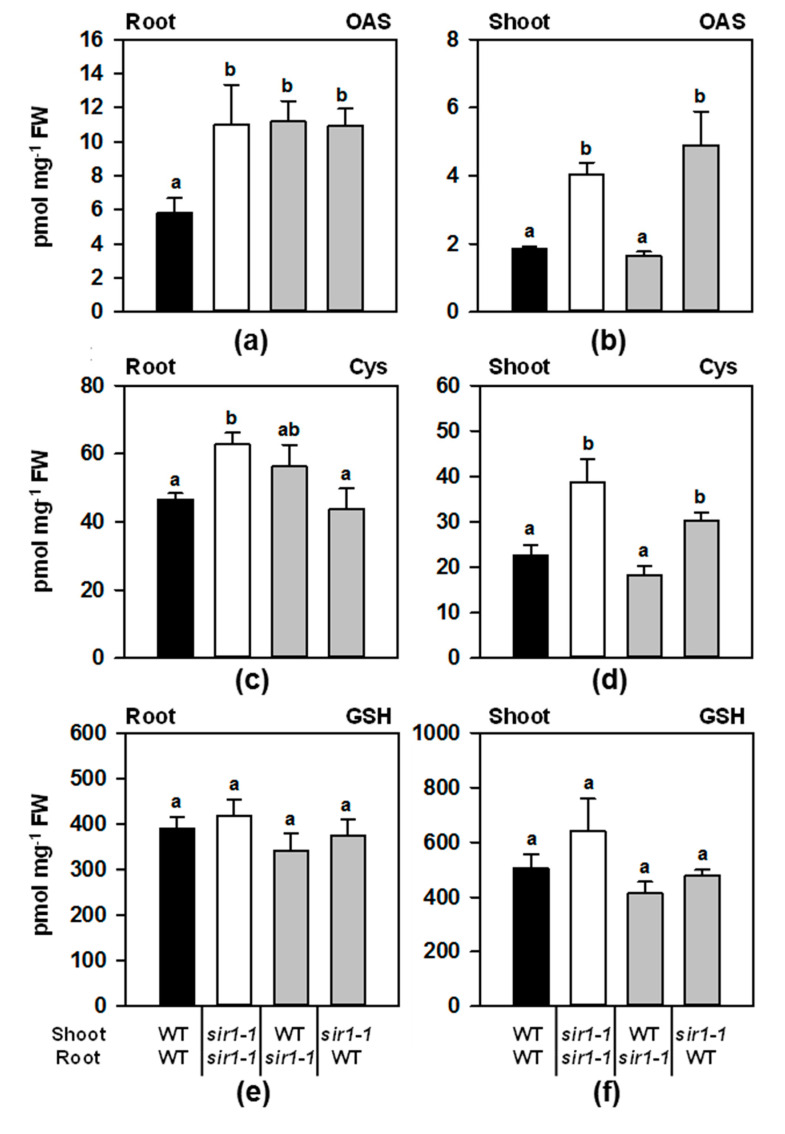
Steady-state levels of OAS and thiols in micrografted plants. Concentrations of OAS (**a**,**b**), Cys (**c**,**d**) and GSH (**e**,**f**) in roots (**a**,**c**,**e**) and shoots (**b**,**d**,**f**). Data represent the means ± SE (*n* = 4). Different letters indicate individual groups identified by multiple pairwise comparisons with a Holm–Sidak, one-way ANOVA (*p*, 0.05) followed by the Student–Newman–Keuls posthoc test.

Surprisingly, the depletion of SiR activity in the roots and the shoots (*sir1-1*/*sir1-1*) had no significant impact on the steady-state levels of sulfate, phosphate or nitrate in the root. Consequently, also in the roots of heterografted plants, sulfate, phosphate and nitrate concentrations remained unaltered (Figure 3a,c,e). Phosphate was not significantly affected in the shoot or root of any grafting combination (Figure 4d), suggesting that the grafting procedure had no general effect on anion uptake. Nitrate concentrations in shoots again mirrored the levels observed in ungrafted plants [12] and showed the opposite pattern compared to sulfate: it was significantly decreased in the shoot of *sir1-1*/*sir1-1* and *sir1-1*/WT plants (1.8-fold and 1.2-fold, respectively) and unchanged in the WT/*sir1-1* shoot (Figure 4f).

Thus, the anion concentration pattern in the organs of micrografted plants appears to be strictly dependent on the shoot genetic background, with the specific accumulation of sulfate and decreased nitrate in the shoots of *sir1-1* background. On the contrary, the depletion of sulfur reduction in the roots of WT/*sir1-1* or *sir1-1*/*sir1-1* has no significant influence on macronutrient accumulation in the shoot.

### 2.4. OAS and Thiols in the Grafted Plants

The cysteine precursor *O*-acetylserine (OAS) is intensively discussed as a signal for the expression of many genes related to sulfur metabolism [15]. The *sir1-1* mutant is characterized by an increased OAS concentration due to the limited flux into cysteine and also an impact on the thiol steady-state level [12]. OAS was found to be increased in the roots of all the grafting combinations (1.9-fold) compared to the WT/WT homografts (Figure 4a). In the shoot, OAS was 2.2-fold accumulated in *sir1-1*/*sir1-1* homografts, thus well reflecting the situation in soil-grown non-grafted *sir1-1* mutant plants [12]. It was unchanged in the WT/*sir1-1* combination and accumulated 2.6-fold in the *sir1-1*/WT combination (Figure 4b). Thus, the *sir1-1* mutation caused the shoot to accumulate OAS even when grafted on a WT root.

The steady-state abundance of cysteine (Cys) largely mirrored the OAS pattern even though Cys concentrations in *sir1-1*/*sir1-1* grafts were not significantly enhanced. The observed 1.7-fold and 1.3-fold increased Cys concentrations in the shoot of *sir1-1*/*sir1-1* and *sir1-1*/WT grafts, respectively, supported the dominating role of the shoot over the root. GSH concentrations were about 10-fold higher compared to Cys levels as could be expected from the analyses of intact plants (see [6], for review). They remained constant within statistical variation in the roots (Figure 4e) and in the shoots (Figure 4f) of all tested grafting combinations.

The quantification of metabolites in the four grafted genotypes allows us to conclude that the depletion of *SiR* gene expression was the primary reason for the specific adaptations of the sulfur and, also to some extent, nitrogen metabolism in the *sir1-1* plant. However, this perturbation of nitrogen metabolism is unlikely to contribute to the observed retarded growth of *sir-1-1*, since (1) total nitrogen content of *sir1-1* is only decreased by 10%, (2) nitrogen-containing biomolecules accumulate in *sir1-1* and (3) the *sir1-1* phenotype can be suppressed by redirecting sulfur-flux from glutathione biosynthesis to cysteine biosynthesis [10,12]. Our findings demonstrate a substantial demand-driven control of nutrient uptake by the roots for supply to the shoot.

## 3. Discussion

Grafting is a well-established method in horticulture for the improvement of woody plants, such as grapevine and apple. Suitable combinations of scion and rootstock can lead to improved plant growth and yield under abiotic and biotic stress [19]. Chimeric plants have been applied to investigate the long-distance transport of large and small molecules via the xylem and the phloem. Remarkably, grafting also contributed to the identification of CEPD1/2 proteins that are shoot-to-root mobile proteins, transmitting the systemic N-deficiency signal. The shoot-specific expression of CEPD1/2 is induced by the root-to-shoot mobile peptide hormone, C-TERMINALLY ENCODED PEPTIDE (CEP), originating from the N-starved roots. This example of antagonistically transported but entailing signals unravels the complexity of organ communication that is critical for coordinating soil-borne nutrient uptake [20]. Such nutrient-deficiency induced long-distance signals include not only peptide hormones and proteins but also nutrients themselves, sugars, hormones, silencing and messenger RNAs (reviewed in [21,22]). Earlier experiments using non-grafting approaches had revealed that sulfur metabolism-related compounds could have long-distance signaling functions. The vascular-transported sulfate for the closing of stomata during early drought stress is one such example [23,24,25], but also phloem-mediated transport of miRNA395 mainly regulating *Sultr2;1* and *APR2* gene expression in roots [26,27], and the source to sink transport of GSH and S-methylmethionine in the phloem may have control functions beyond sulfur supply [28,29]. S-nitrosoglutathione is another sulfur-containing compound that has been suggested to be involved in shoot-to-root communication due to organ-specific dynamic inactivation by S-nitrosoglutathione reductase (GSNOR) [30]. GSNOR rapidly degrades S-nitrosoglutathione and controls the demethylation and expression of transposable elements and stress-responsive genes [31]. Remarkably, DNA demethylation of the *SULTR1;1* promotor also triggers the expression of SULTR1;1 and is responsive to external sulfur supply [32]. GSNOR is particularly important for controlling Fe-metabolism [30]; Fe metabolism is known to be tightly co-regulated with sulfur availability since most of the bound iron is incorporated in iron-sulfur clusters of diverse protein, including SiR (reviewed in [33] and discussion of [34]).

Investigations in *Brassica oleracea* showed that prolonged sulfate starvation led to an increased biomass ratio of root to shoot but revealed limited shoot to root signaling with respect to the expression of sulfate transporters [35]. Thus far, grafting has not been applied to investigate root-shoot relationships in sulfur metabolism but split root experiments indicated extensive communication between well-supplied and sulfate starved roots, possibly via the shoot, with respect to sulfate, OAS and GSH as signaling molecules [36,37,38].

A split-root approach with *Brassica napus* and external sulfate deficiency [36,39,40] showed a demand-driven control of sulfate acquisition and assimilation and identified GSH as the likely phloem-translocated signal from the shoot to the root to integrate the nutritional status of the leaves. A similar approach revealed both local and systemic regulation of sulfur-related gene expression with OAS and sulfate itself as major signal components [38]. Using radiolabeled sulfate, the authors found that sulfate is by far the dominating mobile sulfur compound between shoots and roots. In the derived model, local sulfate supply regulates the expression of the *Sultr 1;1* and *Sultr2;1* genes in the root, and the sulfur status in the shoot modulates the OAS-mediated gene expression response by a ‘passive sulfur status-dependent’ transport of sulfate.

The main differences between the grafting approach applied here, and these split root systems are that local and external deficiencies of inorganic sulfate formed the trigger of transport and responses in the plant. In contrast, the chimeric plants with *sir1-1* genetic background displayed an organ-restricted limitation of reduced sulfur, thus providing a novel view on the communication between shoots and roots. 

The grafting of the *sir1-1* shoot to the wildtype root was sufficient to induce the root plasma membrane-resident high-affinity sulfate uptake system in the presence of external sulfate. This finding provides direct evidence for a demand-driven control of sulfate uptake to optimize sulfate movements between the sink and source organs. Such a demand-driven regulation was postulated for a long time [41]. However, the demand-driven control hypothesis was challenged by the finding that local induction of the root-sulfate uptake system by external sulfur-deprivation was not reverted in plants fed via the leaves with atmospheric sulfide [35]. This earlier study clearly demonstrated that the roots perceive the local sulfur supply and react to this limitation with an induction of the sulfate uptake capacity irrespective of the sulfur supply of the shoot. The here demonstrated shoot demand-driven control is not contradicting this previous conclusion but adds a novel layer of complexity to the maintenance of whole plant sulfur homeostasis when external sulfur is not limiting. 

The shoot demand-driven induction of the root sulfate uptake system did not correlate with locally enhanced steady-state levels of previously identified sulfur-starvation signals (Figure 4). However, this result does not exclude the signaling role of these compounds since we are missing information about the subcellular distribution of these signals. Furthermore, some of these signals might be perceived as ratios with additional specifiers. In the case of OAS, a potential sensor would be the OAS-dissociable cysteine synthase complex, consisting of serine acetyltransferase and OAS(thiol)lyase, which can bind OAS and sulfide and is subject to extensive protein modifications [42,43]. Remarkably, OAS-induced dissociation of the CSC is counteracted by sulfide [44]. The recent identification of the ASTOL1 point mutation in the OAS-binding component of the CSC uncovered the pivotal sensing function of the plastid-localized CSC and its importance for optimizing the stress resilience of plants [45]. However, the CSC also operates in the cytosol and the mitochondria as an OAS-sensor [46,47]. Furthermore, sulfide has been shown to act as a mobile gaseous signal, controlling diverse aspects of plant metabolism [48,49,50]. The concept of transceptors, which are defined as metabolite transporters acting as receptors, would allow the sensing of shoot-to-root transport of sulfur-demand signals without the necessity of substantial fluctuations in steady-state metabolite levels [51]. In this respect, it should be noted that plants SULTR possess a STAS domain that is supposed to have a regulatory function and allows for the physical interaction of membrane-resident SULTRs with OAS-TL [52,53]. The absent correlation of *SULTR1;1* and *SULTR1;2* transcriptions in roots with neither OAS, sulfate, or glutathione steady-state levels was observed earlier [54]. In this study, roots were submitted to a wide diversity of experimental conditions, which uncovered that *SULTR1;2* transcriptions correlated better with the metabolic demand of the shoot and the photoperiod, while the *SULTR1;1* gene predominantly but not exclusively, reacted to the local sulfur supply at the rhizosphere.

## 4. Materials and Methods

### 4.1. Plant Genotypes and Growth Conditions

*Arabidopsis thaliana* mutant plant (*sir1-1*) and wildtype control plants were in the Columbia (Col-0) ecotype. The T-DNA insertion line *sir1-1* was described and verified for homozygosity of the corresponding T-DNA insertion with primers according to [12]. Plants were grown in a growth cabinet with an 8 h/16 h day/night cycle at a light intensity of 120 μmol m^−2^ s^−1^ and 22 and 18 °C, respectively.

### 4.2. Grafting

The grafting of Arabidopsis using silicon tubes followed the method described in [55]. Seedlings were grown on plates containing sterile 1/4 MS media with 1% sucrose and 1.4% agar. The medium was supplemented with 40 mg/L ampicillin to inhibit bacteria growth. Seeds were surface sterilized with 1 mL 70% (*v*/*v*) ethanol and 0.01% Triton X-100 for 20–30 min, then washed at least five times with ddH_2_O and transferred onto plates using a pipette. The grafting was performed 7–8 days after placing the plates vertically into the growth cabinet. Seedlings were cut using a razor blade not lower than midway down the hypocotyls. Shoot and rootstocks were separated and a silicon tube (diameter 0.3 mm, opened using a razor blade and cut in small pieces about 2–3 mm long) was slid over the rootstock. Afterward, the scion was pushed into the tube until it reached contact with the rootstock. The grafted plantlets were placed on a new plate containing fresh media and returned to the growth cabinet. Three to four days after grafting, plantlets were inspected for adventitious root formation on the scion. Those roots were removed by cutting them with a razor blade. Successfully grafted plants were maintained for five weeks in the same growth conditions; after 2.5 weeks, they were moved to a fresh plate. At the end of the 5th week, the shoots and roots were harvested separately using different razor blades and forceps in order to avoid contaminations. The genetic identity of wildtype and *sir1-1* scions and rootstocks after grafting was verified by genomic PCR, as shown in [11].

### 4.3. RNA Extraction and Transcript Levels Analyses

Total RNA was extracted from 50 mg of frozen root material using the peqGOLD Total RNA Kit (peqGOLD, Erlangen, Germany) according to the manufacturer’s instructions. Reverse transcription quantitative PCR (RT-qPCR) analysis was performed using the qPCRBIOSyGreen Mix Lo-ROX (PCR Biosystems) in the Rotor-Gene Q cycler (Qiagen, Hilden, Germany). Gene-specific primers used for RT-qPCR: *APR2*_for CCCGTTCACTTTAGCATCATCGGAG; *APR2*_rev GATCGAACCCATTTGTCTCAGAGAC; *SULTR1;1*_for GCCATCACAATCGC TCTCCAA; *SULTR1;1*_rev TTGCCAATTCCACCCATGC; *SULTR1;2*_for GGATCCAGAGATGGCTACATGA; *SULTR1;2*_rev TCGATGTCCGTAACAGGTGAC; *TIP41-like*_for GATGAGGCACCAACTGTTCTTCGTG; *TIP41-like*_rev CTGACTGATGGAGCTCGGGTCG; *SiR*_for TTGAAAAGGTTGGTCTGGACTAC; *SiR*_rev GGTGTTCCTCCTAGCCAAAC.

### 4.4. Metabolite Analyses

Ions were extracted from 50 mg materials in 0.3 mL ddH_2_O. The extraction was carried out at 98 °C for 30 min under constant shaking. The aqueous extracts were diluted three times with ddH_2_O to a final volume of 300 μL and transferred to HPLC vials. The determination was carried out on an ICS-3000 system (Dionex) with an IonPac AS 11 column and 15–300 mM NaOH (Fluka, Buchs, Switzerland, in ddH_2_O) as eluent. Amperometric detection allows quantitative calculation of the organic acids and inorganic ions and was performed using Chromeleon software 6.7 (Dionex, Germany). °C. For the measurement of thiols and OAS, total metabolites were extracted from 50 mg leaf or root materials with 0.5 mL 0.1 M HCl. The determination of OAS was based on the derivatization with the fluorescent dye AccQ-TagTM. An aliquot of 10 μL HCl extracts was mixed with 70 μL borate buffer (0.2 M, pH 8.8) and 20 μL 3 mg ml^−1^ AccQ-TagTM solution. The derivatization was performed at 55 °C for 10 min. The data were analyzed using the software Empower Pro. To detect thiols, 25 μL HCl extracts were incubated with 245 μL reduction buffer (68 mM Tris, pH 8.3; 0.34 mM DTT; 25 μL 0.08 M NaOH) for 1 h at room temperature in the dark. The reduced thiol groups were derivatized with 0.85 mM of the fluorescent dye monobromobimane at room temperature for 15 min in the dark. An aliquot of 705 μL 5% acetic acid was used to stop the derivatization. The separation of thiols was performed by reversed-phase HPLC on a Nova-PakTM C18, 4.6 × 250 mm column as described in [56]. Thiol-bimane derivatives were detected at an emission wavelength of 480 nm upon excitation at 380 nm. 

### 4.5. Statistical Analyses

Statistical analysis was performed using the software suite SigmaPlot 12.5 (Systat). Different letters indicate individual groups identified by multiple pairwise comparisons with a Holm–Sidak, one-way ANOVA (*p*, 0.05) followed by the Dunn´s or the Student–Newman–Keuls posthoc test.
